# Field and experimental data indicate that the eastern cottontail (*Sylvilagus floridanus*) is susceptible to infection with European brown hare syndrome (EBHS) virus and not with rabbit haemorrhagic disease (RHD) virus

**DOI:** 10.1186/s13567-015-0149-4

**Published:** 2015-02-24

**Authors:** Antonio Lavazza, Patrizia Cavadini, Ilaria Barbieri, Paolo Tizzani, Ana Pinheiro, Joana Abrantes, Pedro J Esteves, Guido Grilli, Emanuela Gioia, Mariagrazia Zanoni, Pier Giuseppe Meneguz, Jean-Sébastien Guitton, Stéphane Marchandeau, Mario Chiari, Lorenzo Capucci

**Affiliations:** IZSLER, Istituto Zooprofilattico Sperimentale della Lombardia e dell’Emilia Romagna “Bruno Ubertini”, Via Bianchi 7/9, 25124 Brescia, Italy; Dipartimento di Produzioni Animali, Epidemiologia ed Ecologia, Università degli Studi di Torino, Via Nizza 52, 10126 Torino, Italy; CIBIO-UP, Centro de Investigação em Biodiversidade e Recursos Genéticos Universidade do Porto, InBio, Laboratório Associado, Campus Agrário de Vairão, Vairão, Portugal; Dipartimento di Scienze Veterinarie e Sanità Pubblica (DIVET), Università degli Studi di Milano, Via Celoria 10, 20133 Milan, Italy; Veterinary Practitioner, Via Negri 26, 29122 Piacenza, Italy; ONCFS, National Hunting and Wildlife Agency, Department of Studies and Research, CS 42355, 44323 Nantes, Cedex 3 France; OIE Reference Laboratory for Rabbit Haemorrhagic Disease at IZSLER Via Bianchi 7/9, 25124 Brescia, Italy; Departamento de Biologia, Faculdade de Ciencias, Universidade do Porto, Porto, Portugal; SaBio - Instituto de Investigacion en Recursos Cinegeticos (CSIC-UCLM-JCCM), Ciudad Real, Spain; INSERM, U892, Universite de Nantes, 44007 Nantes, France; CESPU, Instituto de Investigação e Formação Avançada em Ciências e Tecnologias da Saúde, 4585-116 Gandra, Portugal

## Abstract

The eastern cottontail (*Sylvilagus floridanus*) is an American lagomorph. In 1966, it was introduced to Italy, where it is currently widespread. Its ecological niche is similar to those of native rabbits and hares and increasing overlap in distribution brings these species into ever closer contact. Therefore, cottontails are at risk of infection with the two lagoviruses endemically present in Italy: Rabbit Haemorrhagic Disease virus (RHDV) and European Brown Hare Syndrome Virus (EBHSV). To verify the susceptibility of *Sylvilagus* to these viruses, we analyzed 471 sera and 108 individuals from cottontail populations in 9 provinces of north-central Italy from 1999 to 2012. In total, 15–20% of the cottontails tested seropositive for EBHSV; most titres were low, but some were as high as 1/1280. All the cottontails virologically tested for RHDV and EBHSV were negative with the exception of one individual found dead with hares during a natural EBHS outbreak in December 2009. The cottontail and the hares showed typical EBHS lesions, and the EBHSV strain identified was the same in both species (99.9% identity). To experimentally confirm the diagnosis, we performed two trials in which we infected cottontails with both EBHSV and RHDV. One out of four cottontails infected with EBHSV died of an EBHS-like disease, and the three surviving animals developed high EBHSV antibody titres. In contrast, neither mortality nor seroconversion was detected after infection with RHDV. Taken together, these results suggest that *Sylvilagus* is susceptible to EBHSV infection, which occasionally evolves to EBHS-like disease; the eastern cottontail could therefore be considered a “spill over” or “dead end” host for EBHSV unless further evidence is found to confirm that it plays an active role in the epidemiology of EBHSV.

## Introduction

The eastern cottontail (*Sylvilagus floridanus*) is a lagomorph belonging to the *Leporidae* family, which also includes hares (genus *Lepus*) and the European rabbit (genus *Oryctolagus*). The genus *Sylvilagus* separated from the genera *Lepus* and *Oryctolagus* around 12 million years ago [1-3]. The genetic distances among the three genera are almost the same. *Sylvilagus floridanus* originated in North America and was translocated to various European countries including France (1953), Spain (1980), Switzerland (1982) and Italy, where it was introduced to Piemonte, in Northern Italy, in 1966 and subsequently to many other regions (i.e., Lombardia, Veneto, Emilia-Romagna, Marche, Toscana) for hunting purposes. Currently, the eastern cottontail is widespread in the western part of the Po Valley, and the largest “Italian” population still lives in Piemonte, where it occupies an ecological niche typical of the brown hare (*Lepus europaeus*). The risks of crop damage and possible competition for space and food with other lagomorphs as well as of introduction of highly virulent strains of *Francisella tularensis* have already been highlighted [4-6]. Indeed, due to its high reproductive performance, the cottontail has colonized the hare’s historical range, and currently, interspecific competition may represent an important factor limiting hare populations [7]. In particular, the cottontail is blamed for having caused a drastic decline in hare populations in simplified agro-ecosystems, i.e. in areas with a low biodiversity and landscape heterogeneity, in which the two species are sympatric, competing for daytime refuges and feeding sites [8].

Rabbit haemorrhagic disease (RHD) and European brown hare syndrome (EBHS) are two highly contagious and acute fatal diseases caused by distinct but antigenically correlated caliciviruses [9]. RHD was first reported in 1984 in the People’s Republic of China [10] and subsequently in many other countries throughout the world reviewed in [11]. EBHS was first described in Sweden in 1980 [12,13] and is present only in Europe. Both diseases were first reported in Italy in the late 1980s and have been considered endemic since then [14]. Based on both the results of experimental trials and epidemiological data, RHD and EBHS had been initially considered genus-specific, the former infecting only wild and domestic European rabbits (*Oryctolagus cuniculus*) [15] and the latter both brown hares (*Lepus europaeus*) [16] and mountain hares (*Lepus timidus*). A new RHDV-related pathogenic virus emerged in France in 2010; in the following few years, it spread to Italy, Spain, Portugal and Great Britain [17-20]. Among the specific characteristics of the new virus, which has been named RHDV2 [17], is its ability to infect and cause an EBHSV-like disease in Cape Hares (*Lepus capensis* subsp. *mediterraneus*) in Sardinia [21]. RHDV2 was also identified in an Italian hare (*Lepus corsicanus*) in a single case in Sicily [22]. These two events suggest a possible species jump of lagoviruses between lagomorph genera.

The main aim of the present study was to determine the role of *S. floridanus* as the host, vehicle or reservoir of lagoviruses in Italy. Gregg et al. [23] found that *S. floridanus* does not show clinical signs of RHD when challenged with RHDV, but their study does not give information on a possible subclinical infection. In order to study the susceptibility of *S. floridanus* to RHDV and EBHS, and therefore to better evaluate the role of this species in the epidemiology of pathogenic lagoviruses, we analyzed the results of serological and virological surveys conducted in north-central Italy over a 13-year period. To support field results, we additionally performed experimental trials to assess the reproducibility of both RHD and EBHS in seronegative cottontails. Taken together, the results obtained indicate that cottontails are susceptible to EBHSV but not to RHDV infection.

## Materials and methods

### Epidemiological surveys

**Figure 1:**
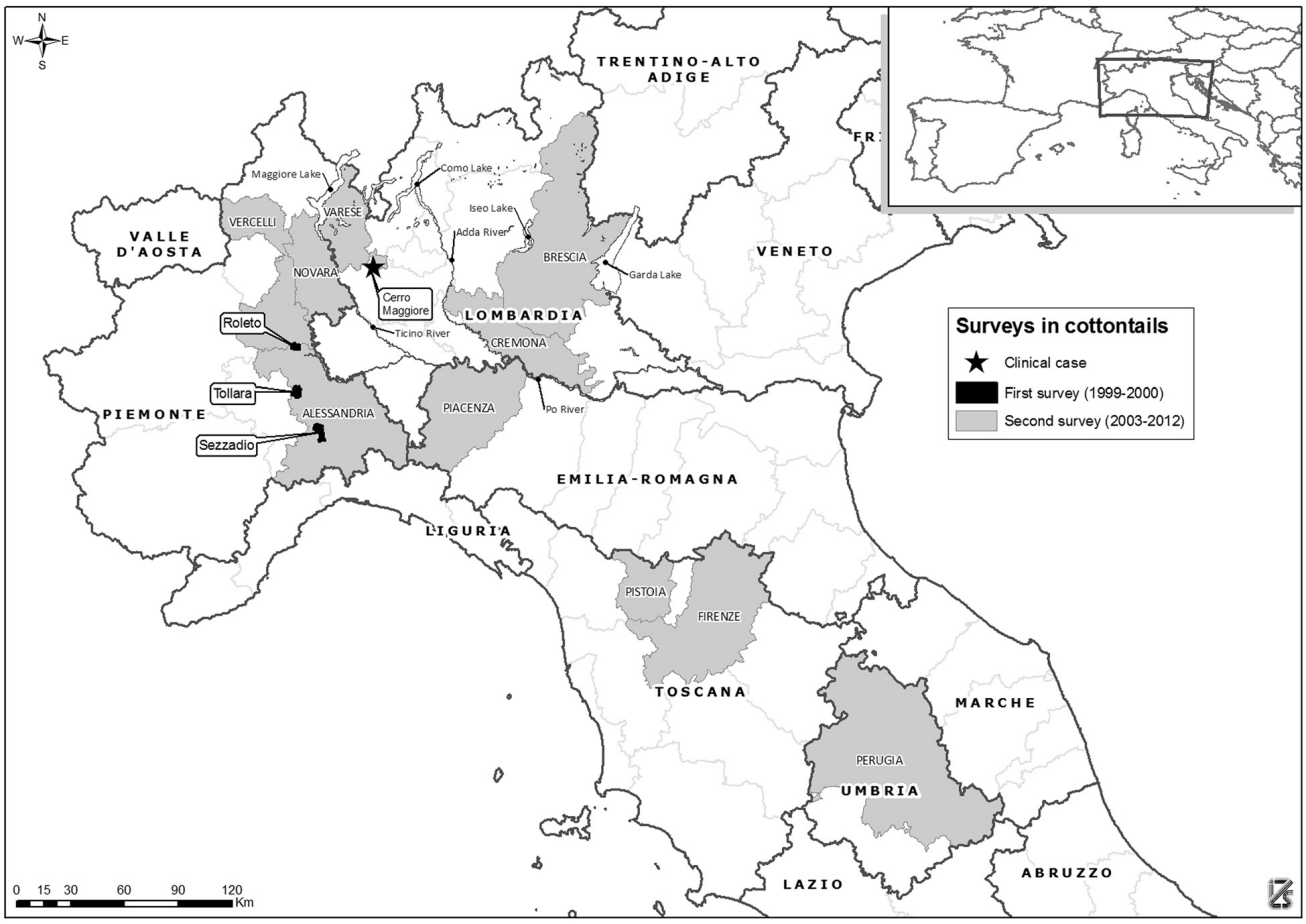
**Maps showing sites in northern and central Italy where cottontails were sampled.** The map shows the sites and areas of north-central Italy where cottontails were sampled during the two epidemiological surveys and where the clinical case of EBHS in cottontails was observed.

During the first survey, we considered three different areas, Roleto, Sezzadio and Tollara, in the Province of Alessandria (lat. 44.916; long. 8.6148) in northwestern Italy (Figure 1). This was an active surveillance monitoring site and the areas were chosen according to the following criteria: i) typical habitat for *S. floridanus*; ii) high density of *S. floridanus* (>50 individuals/km^2^); iii) simultaneous presence of brown hares; iv) prohibition of hunting. We shot approximately 15 individuals in each study area at bimonthly intervals between July 1999 and January 2000 and then again in May and August 2000. In total, we collected blood samples from 252 animals (122 males, 130 females).

The second survey consisted of passive monitoring and surveillance and was conducted from 2003 to 2012. We analyzed 219 blood samples and 108 carcasses from cottontails captured or found dead in 9 provinces of north-central Italy (Figure 1). The number of cottontail carcasses examined each year was highly variable and depended on local surveillance programs (3 on 2006, 23 on 2007, 12 on 2008, 2 on 2009, 50 on 2011 and 18 on 2012).

### Clinical case

In late 2009, an EBHS outbreak occurred in a fenced-in area of approximately 1 km^2^ at Cerro Maggiore (+45° 34′ 55.79″, +8° 58′ 31.52″) north of Milan (Figure 1), where a high density of hares (25 individuals/km^2^) and cottontails (>50 individuals/km^2^) was present. Over a ten-day period (24 December 2009 – 3 January 2010), several hares and some cottontails were found dead, but due to post mortem alterations and predator activity, the carcasses of only two hares and one cottontail were brought to the laboratory for examination. After necropsy, we examined the organs for EBHSV using conventional virological methods. One year later (January 2011), we revisited the same area to assess the serological status of surviving cottontails and hares by taking, respectively, 11 and 25 blood samples from their ear veins.

### Serological analysis

To detect antibodies against RHD and EBHS, we analyzed the serum samples using two specific competitive ELISA (cELISA). These tests, one for each virus (RHDV and EBHS), were developed in-house at the OIE Reference Laboratory, and the procedures are extensively described in the “OIE Manual of Diagnostic Tests and Vaccines for Terrestrial Animals” [24]. The tests are based on competition for the antigen between anti-RHDV or anti-EBHS antibodies adsorbed to the solid phase and those possibly present in the serum sample added to the plate well. The antigen (RHDV or EBHSV) was used at a fixed and pre-determined concentration [14,23-26]. Such ELISA have a high specificity because they primarily measure antibodies binding the antigenic determinants forming the viral surface. Regarding the data interpretation, because this was the first time a serological approach was applied with *S. floridanus*, we used the same cut-off value for rabbits and hares. The sera were classified as negative if the absorbance value of the first dilution (1/10) decreased by less than 20% of the reference value (i.e. the first dilution of the negative control serum) and positive if the absorbance value decreased by 30% or more. Sera with a decrease between 20% and 30% were considered equivocal. The titre of positive sera corresponded to a dilution yielding an absorbance value of approximately 50% of the value of the negative control serum. In order to detect anti-EBHSV and anti-RHDV IgM in the sera of experimentally infected animals, we used two similar sandwich ELISA developed using anti-isotype MAbs (IgM-ELISAs) after having ascertained that *S. floridanus* IgM were also recognized by the test [24].

### Virological analysis

Carcasses and/or organs from the hares, rabbits and cottontails collected during the second survey, from a clinical case, and from experimental trials were submitted to necropsy and virological examination for EBHS and/or RHD. In particular, the liver and spleen were examined using methods commonly employed in routine laboratory diagnostics, i.e., sandwich ELISA and RT-PCR. These are also “in house” tests, fully described in the “OIE Manual of Diagnostic Tests and Vaccines for Terrestrial Animals” [24].

### Genomic analysis

Total RNA was isolated from liver homogenates of one hare and one cottontail using the Trizol reagent (Invitrogen) according to the manufacturer’s instructions. Reverse transcription (RT) was carried out with an oligo(dT) primer using the High Capacity cDNA Reverse Transcription Kit (Applied Biosystem) according to the protocol suggested by the supplier. The gene encoding the capsid protein VP60 was amplified by PCR in two overlapping regions (PCR1-959 bp: EBHS-F2 5’-CTGGAATATGAATGGTGAAACC-3’, EBHS6-R 5’- GAGCGCTGCTCAACGTAGAA-3’; PCR2-915 bp: EBHS5-F 5’- CGACAGGAAGAGGATCGTCT-3’, EBHSV-R7 5’-AAACCTGGGGCTGGACCAGC-3’). Cycle conditions were 5 min at 95 °C followed by 40 cycles of 30 s at 95 °C, 30 s at 58 °C, 1 min at 72 °C and a final extension of 7 min at 72 °C. The PCR products were purified from agarose gel and sequenced twice in both directions with the PCR primers by an automated fluorescence-based technique according to the manufacturer’s instructions (ABI-PRISM 3130 Genetic Analyzer, Applied Biosystems); they were assembled with SeqMan Pro (DNASTAR, Lasergene 10).

The resultant sequences were put into the local alignment search tool (BLAST) in the GenBank library to confirm the specificity of a positive reaction and to estimate the degree of identity with the detected strain. The sequences obtained were aligned with the available GenBank sequences using ClustalW, as implemented in MEGA 5.1 [27].

Due to the phenotypic similarities existing between cottontails and brown hares, especially when they are young and sub-adult, and in consideration of the exceptionality of the case observed, it was decided to confirm the genetic identity of the studied animals as *Sylvilagus* sp.. Therefore a set of discriminating genetic markers was amplified by PCR. The following have been shown to have specific SNP for *Oryctolagus*, *Sylvilagus* and *Lepus* genera: CXCR4 [28], CCR5 [29], IGHG hinge region [30] and IGHG CH2 [31]. The mitochondrial Cytb was also analyzed. Total genomic DNA was extracted from frozen liver tissue using the EasySpin Genomic DNA Minipreps Tissue Kit (Citomed). PCR amplification of the set of nuclear and mitochondrial markers was conducted using primers designed on available sequences from *Oryctolagus cuniculus* or *Sylvilagus* sp. (GenBank Acession Numbers: CCR5: DQ142884; CXCR4: EU258276; IGHG: AY386696; CytB: HQ143462). The PCR primer pairs for each genetic marker, amplified fragment length and annealing temperatures are indicated in Table 1. The PCR reaction was performed with 0.5 μL of DNA in a final volume of 5 μL containing 2.5 μL of HotStarTaq DNA Polymerase Multiplex PCR Master Mix (Qiagen) and 2 pmol of each oligonucleotide. Cycle conditions were 15 min at 95 °C followed by 35 cycles of 30 s at 95 °C, 30 s at the annealing temperature and 45 s at 75 °C (see Table 1 for the annealing temperatures). A final extension was carried out for 20 min at 60 °C. After purification, PCR products were sequenced on an automatic sequencer (ABI PRISM 310 Genetic Analyzer, PE Applied Biosystems) using the same PCR primers.

**Table 1:** **Primers used for PCR amplification and sequencing**

**Genetic marker**	**Primer**	**Primer sequence (5′-3′)**	**Amplified fragment length**	**Annealing temperature (°C)**
CCR5	CCR5F	TCTACCTGCTCAACCTGGCCA	574	58
	CCR5R	TGTTGGAGCTGCTGCAGTTA		
CXCR4	CXCR4F	CATCTTCTTGACTGGCATAG	804	49
	CXCR4RJ1	GCGTTAGCTGGAGTGAAAA		
IGHG hinge region	FG12	TCAGGCCCAGACTGTAGACC	680	60
	RE	ACGGTCCCCCCAGGAGTTCA		
IGHGCH2	F3	GTGAGTCCCATTAGCCTCAC	830	60
	RG31	TTGGAAGGAATCAGGACAGC		
CytB	CytBF	TTCAAATCCTAACCGGCCTA	500	47.5
	CytBR	AACCTAGGGCGTCCTTAATT		

The obtained sequences were aligned with published sequences available in GenBank using ClustalW [32] as implemented in the BioEdit software [33] and adjusted by visual inspection. For each genetic marker studied, the denomination and GenBank accession number of the nucleotide sequences are reported in Tables 1 and 2.

**Table 2:** **Genetic distances between the studied individual and published Sylvilagus, Oryctolagus and Lepus nucleotide sequences**

	***Sylvilagus***	**Sample**	***Oryctolagus***	**Genbank accession numbers**
**CCR5**				
*Sylvilagus*	----	----	----	DQ017768
Sample	0.009	----	----	KF620521
*Oryctolagus*	0.056	0.056	----	DQ142884
*Lepus*	0.047	0.045	0.059	DQ146763
**CXCR4**				
*Sylvilagus*	----	----	----	EU258272
Sample	0.001	----	----	KF620522
*Oryctolagus*	0.009	0.010	----	EU258276
*Lepus*	0.011	0.012	0.011	EU258265
**IGHG hinge region**				
*Sylvilagus*	----	----	----	DQ206979
Sample	0.015	----	----	KF620519
*Oryctolagus*	0.038	0.045	----	DQ206981
*Lepus*	0.068	0.075	0.060	DQ206976
**IGHG CH2**				
*Sylvilagus*	----	----	----	AJ295223
Sample	0.017	----	----	KF620520
*Oryctolagus*	0.048	0.036	----	AJ430862
*Lepus*	0.043	0.031	0.038	AJ295217
**Cyt B**				
*Sylvilagus*	----	----	----	HQ143462
Sample	0.002	----	----	KF620523
*Oryctolagus*	0.150	0.152	----	HQ596486
*Lepus*	0.144	0.146	0.144	AY745113

For each genetic marker, pairwise distances were calculated in MEGA5.1 [27] using the options p-distance and complete deletion of positions with gaps or missing data.

### Experimental reproducibility of RHD and EBHS in cottontails

To assess the reproducibility of RHD and EBHS in cottontails, we used seven conventional rabbits (New Zealand hybrids) of approximately four months of age, two captive-reared sub-adult brown hares and ten cottontails, which were live-trapped and then brought to the animal facility of IZSLER in Brescia. All animals were housed individually in cages in three isolation units. The negative serological status of each animal was verified by a blood sample taken just prior to infection.

The RHDV used for infection was our laboratory’s reference strain, RHDV-BS89. The experimental infection contained approximately 10^5^ lethal doses (LD_50_)/mL [34], Mortarino, personal communication, 1990. The EBHS isolate (i.e., EBHS reference strain EBHSV-BS89, GenBank accession number X98002) derived from a severe outbreak of EBHS in naturally infected, captive hares. The inoculum corresponded to a 10% (w/v) liver suspension in PBS, and its infectivity and virulence have been assessed in previous experiments [16]. The inocula were diluted in sterile PBS (EBHSVBS89 = 1:2, and RHDVBS89 = 1:10) and filtered on a 0.2 μm Millipore filter before being used. To ensure that the recorded serological results were in fact due to RHDV replication, we inactivated a few mL of RHDV inoculum with 1% formaldehyde for 24 h at 25 °C.

In the first trial, two cottontails and three rabbits were infected with a 2 mL dose of RHDV-BS89 via the oro-nasal route, two cottontails and two rabbits were administered an inactivated inoculum by the same route, and the remaining two cottontails and two rabbits were maintained as uninfected controls. The animals were kept in three separate rooms, divided according to the type of treatment; they were assessed daily for mortality or the presence of clinical signs over a two-week period. Blood samples were taken at 14 and 28 days post infection (pi) for serological analysis of both specific anti-RHDV antibodies and IgM isotype antibodies. In the second experiment, four seronegative cottontails and two seronegative hares were infected via the oro-nasal route with 2 mL of EBHSV-BS89. The animals were kept in separate cages in the same room, checked daily for the presence of clinical signs and mortality for two weeks and serologically controlled for both specific anti-EBHSV antibodies and IgM isotype antibodies at eleven and 32 days pi.

Experiments were carried out in accordance with the European Communities Council Directive of 24 November 1986 (86/609/EEC) and with National laws and regulations regarding the care and use of animals. At the end of the experiments, the surviving animals were humanely euthanized.

## Results

### Epidemiological surveys

**Figure 2:**
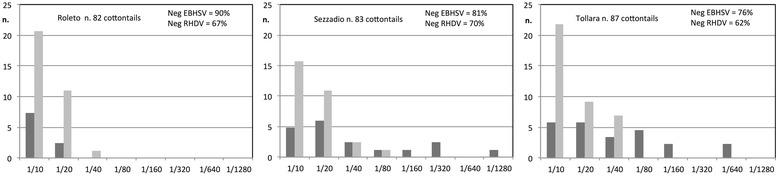
**Results of serological analysis for RHD and EBHS antibodies in cottontails during the first survey.** Results of serological analysis for RHD and EBHS in cottontail sera from three areas in the Alessandria province (black columns = AbRHDV; grey columns = AbEBHSV).

The results of the serological survey performed in 1999–2000 in three different areas of the province of Alessandria are reported in Figure 2. Overall, in serum samples from cottontails, seroprevalences of 17.9% and 33.7% were observed for EBHSV and RHDV antibodies, respectively. These values were reduced to 11.9% and 14.3%, respectively, when considering titres equal to or higher than 1/20. A number of cottontails sera exhibited high titres for EBHSV (i.e., up to 1/1280); in contrast, the highest RHDV titre was only 1/80 and was detected in only one serum sample. Twenty-nine sera were positive for both viruses (65% and 34% of EBHSV and RHDV positive sera, respectively). In 14 of them, the EBHSV titres were higher than the RHDV ones and in the remaining 15 samples, RHDV titres were slightly higher or the titres were similar (1/10-1/20) for the two viruses. In contrast, positive samples were not uniformly geographically distributed. In fact, all serum samples collected in one area (Roleto) were negative or <1/20 for both EBHSV and RHDV antibodies; indeed, no positive samples were found for RHDV during the visits in January and for EBHSV during two visits (September and January) (data not shown). In addition, we observed that the seroprevalence of EBHS in the two areas that had positive sera (i.e., Sezzadio and Tollara) increased with time (data not shown). In particular, at Tollara, all the sera sampled in July 1999 were negative, but the prevalence increased to 26.7% in September 1999 and to 33.3% in both November 1999 and January 2000, with titres as high as 1/40, 1/80 and 1/640, respectively.

During the period from 2003 to 2012, following the implementation of surveillance programs in north-central Italy, 219 blood samples were taken from cottontails in nine provinces. The results of the serological analysis for EBHSV antibodies are detailed in Table 3. The overall prevalence was 20.1%; in particular, 16.0% of animals tested had low titres (1/10–1/20); an almost equal percentage exhibited medium (1/40–1/160 = 2.2%) or high titres (1/320 to 1/640 = 1.9%), and the remaining samples (79.9%) were negative. During this second survey, we also received and examined 108 carcasses of cottontails, captured or found dead. After necropsy, regardless of the presence of specific lesions, the target organs (i.e., liver and spleen) were examined to detect lagoviruses (i.e., EBHSV and RHDV). All carcasses were negative for both lagoviruses.

**Table 3:** **Results of serological analysis for EBHS in cottontail sera during the second survey (2003–2010)**

**Year titre**	**2003**	**2004**	**2005**	**2006**	**2007**	**2008**	**2009**	**2010**	**2011**	**2012**	**TOTAL**
Neg.	7	8	30	7	56	11	1	10	28	17	175
1/10	0	0	7	5	1	3	0	0	11	1	28
1/20	0	0	2	2	0	0	0	0	3	0	7
1/40	0	0	1	0	1	1	0	0	0	0	3
1/80	0	0	0	1	0	1	0	0	0	0	2
1/160	0	0	0	0	0	0	0	0	0	0	0
1/320	0	0	0	0	0	0	1	0	0	0	1
1/640	0	0	0	1	0	2	0	0	0	0	3
1/1280	0	0	0	0	0	0	0	0	0	0	0
Total	7	8	40	16	58	18	2	10	42	18	219

### Clinical case

**Figure 3:**
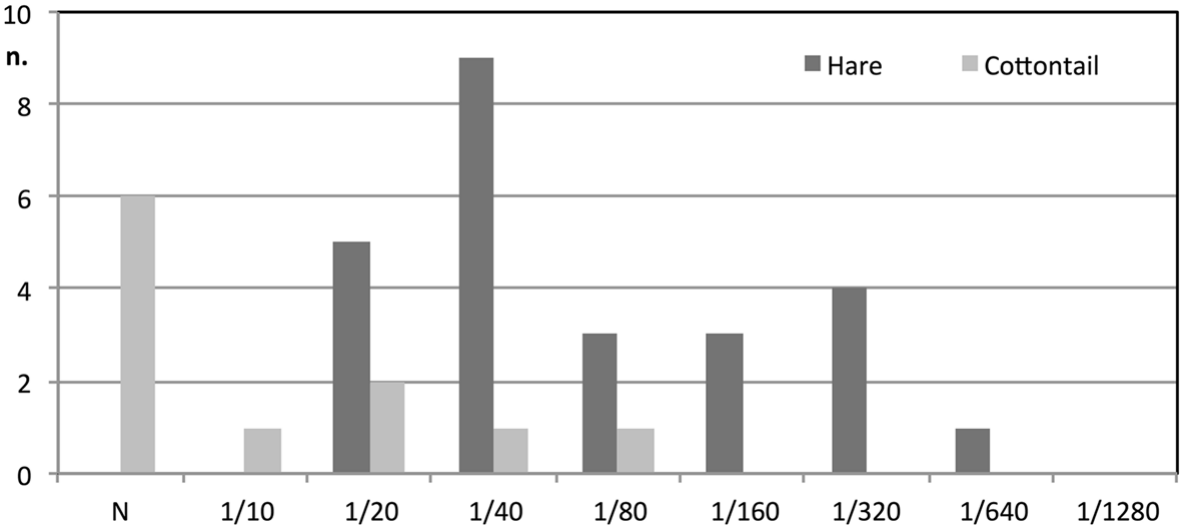
**Results of serological analysis for EBHS in hare and cottontail sera after the clinical case.** Results of serological analysis for EBHS in hare (*n* = 25) and cottontail (*n* = 11) sera after the outbreak in Cerro Maggiore in 2011 (black columns = hares; grey columns = cottontails).

At necropsy, the lesions detected in the two hares and the single cottontail found dead during the outbreak exhibited lesions typical of EBHS: congestion of tracheal mucosa, petechial lung haemorrhages, friable, fatty and discoloured liver with an accentuated lobular pattern, and congestion and enlargement of the spleen. All these animals tested virologically positive for EBHSV in the liver and spleen. The results of the serological assessment undertaken one year after the outbreak are reported in Figure 3. All analyzed hares were positive and showed an average antibody titre higher than that of cottontails. Of the eleven cottontails sampled, five (45.5%) had titres between 1/10 and 1/80 and six (54.5%) tested negative.

Because the necropsied animals (i.e., two hares and one cottontail) were naturally infected by EBHS, the identified strains from both species were amplified, sequenced and compared. The capsid protein (VP60) amplified by RT-PCR from the cottontail was completely sequenced (GenBank JX195102) and compared to the sequence of virus isolated from one hare found dead in the same area and period (GenBank KF591083)*.* The nucleotide identity of the two strains was 99.9%, and the amino acid identity was 100%, indicating the presence of the same viral strain in the two species. The sequences obtained for all five amplified genetic markers were closer to published *Sylvilagus* sequences than to *Oryctolagus* or *Lepus* sequences. Analysis of the variable sites for each genetic marker revealed a few positions that differed between the published *Sylvilagus* sp. sequences and our sample; there was consistently more variation between *Sylvilagus* sp. and *Oryctolagus* or *Lepus* published sequences. Consistently, for all analysed genetic markers, the genetic distances were significantly lower for the *Sylvilagus* sample pair (0.001–0.017) than for *Oryctolagus* sample (0.010–0.152) or *Lepus* sample (0.012–0.146) pairs (Table 2). Thus, the genetic markers analysed confirm that the studied individual was a *Sylvilagus* and thus it had been correctly identified on the basis of its gross appearance.

### Experimental reproducibility of EBHS and RHD in cottontails

The inoculation of RHDV caused an acute form of RHD in the three seronegative rabbits, which all died within 72 h pi and exhibited typical signs and lesions. RHDV positivity was confirmed by a sandwich ELISA test (data not shown). All remaining animals, i.e., all the cottontails and those rabbits inoculated with inactivated RHDV or kept as uninfected controls, remained healthy during the 14 days of observation. Their sera were collected at 14 and 28 days pi, and no antibodies, either IgM or total specific antibodies, were detected using RHDV ELISA (data not shown).

During the second trial, which involved inoculation with EBHSV, one cottontail out of four died at four days pi, and at necropsy it had typical gross lesions attributable to EBHS. The identification of EBHSV was confirmed by virological ELISA. In the sera of the two hares and three cottontails that survived, we detected seroconversion with IgM titres up to 1/40 000 and total specific antibodies up to 1/1280.

## Discussion

Cottontails were first introduced in Italy in 1966 and then in France in 1972. Massive introductions were then made from the late 1970’s to the late 1980’s mainly in Italy and France but also in Switzerland, Spain and Luxemburg [35]. Following these introductions, cottontails established in Italy, where populations progressively increased from the late 1970s to the late 1980s. During the same period, two new diseases, RHD and EBHS, emerged within the European lagomorph species *O. cuniculus* and *L. europaeus* in China and northern Europe, respectively. RHD and EBHS are similar; they are acute and lethal forms of hepatitis caused by two distinct but related lagoviruses. RHDV infects wild and domestic rabbits whereas EBHSV infects hares, both in the wild and in captivity. They are responsible for high mortality rates and account for a strong economic burden on the rabbit and hare industries and on game animal management.. Therefore, this study was designed to better investigate the role of cottontails (*S. floridanus*) in the epidemiology of lagoviruses and in particular, to ascertain whether this species could be infected by any of the known pathogenic lagoviruses, i.e., RHDV or EBHSV. Overall, the results of two consecutive epidemiological surveys, experimental trials and observations of a natural outbreak of EBHS in sympatric hares and cottontails are indicative of natural susceptibility among cottontails to EBHSV, albeit with only sporadic cases of clinical disease.

Our first approach was to evaluate the serological results obtained from cottontail sera using the same ELISA parameters (i.e., threshold values and titre computation) that are employed for serology of RHD in rabbits and EBHS in hares. A general analysis of the data indicates that these parameters could be considered acceptable for cottontail serology as well. In fact, in cottontails experimentally infected with EBHSV, serological titres reached high values within a few days and the kinetics were similar to those induced by EBHSV in hares. These results suggest that the initially chosen threshold ELISA values, identical to those established for hares, were appropriate for cottontails as well. Therefore, the serological results obtained from cottontails can be considered reliable.

The results of the first serological survey indicate that approximately 20% of the tested cottontails may have been naturally exposed to EBHSV; the high EBHSV titres found in some seropositive cottontails were comparable to those detected in the sera of RHD-convalescent rabbits [24,36] and EBHS-convalescent hares [14,37]*.* In addition, we observed that the seroprevalence of EBHSV in the areas of Sezzadio and Tollara, where high titres were found, increased with time, in agreement with the epidemiological characteristics of EBHS that indicate a strong seasonal peak in incidence in the autumn [14]. With regard to RHDV serology, the titres detected were always too low to be considered directly induced by RHDV or by a cross-reaction with EBHSV-induced antibodies. However, the prevalence of low titre RHDV-positive sera was higher than that for EBHSV. This result strongly suggests, as a possible explanation, the presence in cottontails of a non-pathogenic lagovirus genetically related to those found in rabbits [38].

The results of the second serological survey conducted across a ten-year period confirmed the presence of specific EBHSV antibodies among cottontail populations in north-central Italy. The prevalence found in cottontails was similar to that commonly detected in hares hunted in low-density hare populations in northern Italy [39,40].

The experimental trials confirmed that cottontails are not susceptible to experimental infection with RHDV, as previously observed [22], but clearly demonstrated that EBHSV can infect *S. floridanus* via the oro-nasal route that corresponds to the natural way of transmission. In addition, a detectable level of seroconversion was observed in the surviving animals (i.e., three cottontails and two hares), comparable with the levels detected in convalescent hares in natural outbreaks [14,37,39]. The fact that the two experimentally infected hares did not die of EBHS is not unexpected; the mortality rate observed in previous experiments was quite variable, ranging from zero to 50% [Lavazza and Capucci, personal data].

In late 2009, the occurrence of one natural case of EBHS in sympatric populations of hares and cottontails living in a fenced-in area strongly supported these results. Several hares and cottontails were found dead during the outbreak; two hares and one cottontail, delivered to the laboratory, tested positive for EBHSV, and the genomic comparison between the strains identified from both species revealed 100% VP60 amino acid identity, indicating the presence of the same EBHSV strain in the two species. In addition, a serological survey conducted in the same area one year later revealed that approximately half the *Sylvilagus* individuals had had contact with the virus, as indicated by low-medium antibody titres.

Considering the outcome of field surveys and experimental trials we may conclude that cottontails appear to be susceptible to EBHSV infection and are likely more resistant to the development of overt disease than hares. In fact, their susceptibility to EBHSV does not appear to be a problem for the conservation of this invasive species; no outbreaks were recorded in free-living populations other than the one in late 2009. However, by being infected with EBHSV, cottontails may play a certain role in the spread of the virus. We demonstrated that in nature cottontails become infected but only sporadically develop the disease. EBHSV circulates in cottontails but the serological results could also arise if the virus spreads through a sympatric hare population and the cottontails become simply affected as “spill-over” or “dead end” host. Since we do not have a reliable estimate of the prevalence nor do we know whether the infection is dose-dependent or to what extent the viral infectivity and spread in the field are amplified, we cannot confirm that cottontails play an active role in the epidemiology of EBHSV unless further evidence is found.

The susceptibility of *S. floridanus* to EBHSV broadens our perspective on its epidemiological role in lagovirus diseases. The fact that cottontails support EBHSV replication could influence the evolution and outcome of EBHS in hares at a local level and could act as an example of “parasite mediated competition” [41]. In fact, according to the proposed epidemiological model of spread of this disease [38], the ability of EBHSV to infect and/or kill hares in endemic areas is inversely proportional to their population densities. Therefore, since cottontails living in sympatry with hares are quite common in some European countries, the EBHS model should also consider the impact of this species. In particular, cottontails likely act as a carrier of EBHSV and may favour the introduction, persistence and spread of the virus and the occurrence of EBHS cases. This may be especially true in open hunting areas, characterized by simplified agro-ecosystems, which cannot sustain stable, high density hare populations even on a short time scale [7].

In conclusion, further studies are needed to verify the incidence of EBHS infections in sympatric populations of hares and cottontails and to better define, either experimentally or in natural outbreaks, the characteristics (i.e., morbidity, mortality rates, age of affected animals, gross lesions, seroconversion, etc.) of the disease in cottontails. In addition, it is necessary to search for lagovirus genome sequences in organs of healthy cottontails using sensitive molecular methods to ascertain whether they are hosts of non-pathogenic lagoviruses. Finally, considering the capacity of the recently identified RHDV2 to infect species other than the European rabbit, the possibility that RHDV2 can infect or cause disease in *S. floridanus* should be verified, either experimentally or through field observations.

The cottontail is widespread in northern Italy, and any eradication action, even if recommended by the Council of Europe (Recommendation no. R 85–14, 1985), should be considered unfeasible. Considering the adaptability of this species, a further expansion in Northern Italy is likely to occur in the coming years and its population density is likely to increase [42]. Given this context, the possible health interactions with native hares, especially in sympatric populations, must be carefully evaluated through the application of a continuous surveillance program.

## Abbreviations

RHD: Rabbit haemorrhagic disease
EBHS: European brown hare syndrome
cELISA: Competitive ELISA
MAbs: Monoclonal antibodies
IgM-ELISAs: Anti-isotype MAbs sandwich ELISAs
LD_50_: Lethal dose
pi: Post infection
